# Assessing frailty amongst older people admitted to hospital in a low-income setting: a multicentre study in northern Tanzania

**DOI:** 10.1186/s12877-024-04789-6

**Published:** 2024-02-26

**Authors:** Sean L Davidson, Luke Emmence, Sara May Motraghi-Nobes, Emily Bickerstaff, George Rayers, Godrule Lyimo, Joseph Kilasara, Mary Chuwa, Fortunatus Kisheo, Elibariki Kisaruni, Sarah Urasa, Emma Mitchell, Catherine L Dotchin, Richard W Walker

**Affiliations:** 1 Newcastle University, Newcastle Upon Tyne, UK; 2 Northumbria Healthcare NHS Foundation Trust, Morpeth, UK; 3 Kilimanjaro Christian Medical Centre, Moshi, Tanzania; 4 Kilimanjaro Christian Medical University College, Moshi, Tanzania; 5 Mawenzi Regional Referral Hospital, Moshi, Tanzania; 6 Hai District Hospital, Moshi, Tanzania; 7 Machame Lutheran Hospital, Moshi, Tanzania; 8 North Bristol NHS Foundation Trust, Bristol, UK

**Keywords:** Frailty, Frail, Old adults, LMIC, Low- and middle-income countries, Low-income countries, Tanzania, Sub-saharan Africa, Hospital inpatients

## Abstract

**Background:**

Populations are ageing globally and Low- and Middle-Income Countries (LMICs) are experiencing the fastest rates of demographic change. Few studies have explored the burden of frailty amongst older people in hospital in LMICs, where healthcare services are having to rapidly adapt to align with the needs of older people. This study aimed to measure the prevalence of frailty amongst older people admitted to hospital in Tanzania and to explore their demographic and clinical characteristics.

**Methods:**

This study had a prospective observational design. Over a six-month period, all adults ≥ 60 years old admitted to medical wards in four hospitals in northern Tanzania were invited to participate. They were screened for frailty using the Clinical Frailty Scale (CFS) and the Frailty Phenotype (FP). Demographic and clinical characteristics of interest were recorded in a structured questionnaire. These included the Barthel Index, the Identification of Elderly Africans Instrumental Activities of Daily Living (IADEA-IADL) and Cognitive (IDEA-Cog) screens, the EURO-D depression scale and Confusion Assessment Method.

**Results:**

540 adults aged ≥ 60 were admitted, and 308 completed assessment. Frailty was present in 66.6% using the CFS and participants with frailty were significantly older, with lower levels of education and literacy, greater disability, greater comorbidity, poorer cognition and higher levels of delirium. Using the FP, 57.0% of participants were classed as frail though a majority of participants (*n* = 159, 51.6%) could not be classified due to a high proportion of missing data.

**Conclusions:**

This study indicates that the prevalence of frailty on medical wards in northern Tanzania is high according to the CFS. However, the challenges in operationalising the FP in this setting highlight the need for future work to adapt frailty screening tools for an African context. Future investigations should also seek to correlate frailty status with long-term clinical outcomes after admission in this setting.

**Supplementary Information:**

The online version contains supplementary material available at 10.1186/s12877-024-04789-6.

## Background

Populations are ageing globally and rates are particularly prominent in Low- and Middle-Income Countries (LMICs) where an expected 80% of the world’s older people will reside by 2050 [1]. Sub-Saharan Africa (SSA) is amongst the regions experiencing the fastest rates of demographic change [1]. In Tanzania, between 2000 and 2020, life-expectancy at birth rose from 50.8 to 65.8 years [2]. These gains represent an extraordinary success story. However, despite longer life, many older Tanzanians report poor health, quality of life and well-being in older age [3]. Similar challenges are faced across SSA, and indeed in many other LMICs around the world, where healthcare systems have had little time to develop services which align with the needs of older people [4, 5].

There is no single consensus definition of frailty, however it may broadly be characterised as a state of poor health and reduced resilience that is related to age, but not an inevitable consequence of ageing [6–8]. A range of tools exist for its measurement, two of the most widely utilised being Rockwood’s Clinical Frailty Scale and Fried’s Frailty Phenotype [6, 9, 10]. Different conceptions of frailty underpin these instruments, and consequently they (and the myriad of other available tools) produce differing estimates of prevalence [11]. Irrespective of which tool is used, in studies from High-Income Countries (HICs), older adults living with frailty who are admitted to hospital experience longer admissions, greater functional decline and higher mortality [12]. Comprehensive geriatric assessment (CGA) is considered the gold-standard model of care for the diagnosis and treatment of frailty [13]. However, it is resource intensive, requiring geriatricians and a range of allied healthcare professionals. Healthcare systems in SSA have considerable constraints on personnel and resources, and very few physicians with specialist training in geriatric medicine [14]. Consequently, tools for the identification of frailty in these environments must be rapid, require minimal equipment and be designed for use by the non-specialist.

Previous work has explored frailty in a community setting in Tanzania and demonstrated the cultural relevance of the frailty construct [15, 16]. To-date, only two studies have looked at the prevalence of frailty amongst hospital inpatients in SSA, both using the Clinical Frailty Scale (CFS). In the first, Adebusoye et al. found that 63.3% of 450 patients aged over 60 years admitted to medical wards in a tertiary hospital in Nigeria were frail [17]. In the second, Leopold-George et al. found 22% of 299 surgical inpatients aged 18–90 admitted to three academic hospitals in South Africa were frail [18]. It is important to emphasise that the CFS is not validated for use in younger people, limiting the generalisability of the results from the latter study.

The aims of the present study were to measure the prevalence of frailty amongst older people admitted to hospital in northern Tanzania and to explore their demographic and clinical characteristics. Two different commonly used frailty instruments were utilised to aid comparisons with existing literature. Furthermore, based on established reports, it was anticipated that people living with frailty would be more likely to be older, female, unmarried, to have lower levels of education, a greater number of chronic diseases, a greater burden of disability, more depressive symptoms and poorer cognition [15, 17, 19, 20].

## Methods

### Setting

This study was conducted on medical wards in four hospitals in northern Tanzania that were selected purposively to reflect the services available to older people in the region, which are organised according to escalating levels of referral from District, to Regional and Zonal levels. This included hospitals in urban, sub-urban and rural areas, with a mixture of government and privately run facilities:

1. Kilimanjaro Christian Medical Centre (KCMC), a Zonal level and University hospital located on the outskirts of the town of Moshi, owned by a faith-based organisation. The participating male and female medical wards had a total bed capacity of 75.
2. Mawenzi Regional Referral Hospital (MRRH), a large government-run Regional level hospital in the centre of Moshi. Participating male and female medical wards had 49 beds.
3. Hai District Hospital (HDH), a rapidly expanding government-run District level facility in the small but growing town of Boma Ng’ombe. Male and female wards had a total of 60 beds.
4. Machame Lutheran Hospital (MLH), a small District level hospital in a rural setting in the foothills of Kilimanjaro, operated by a faith-based organisation. Participating wards had a capacity of 36 beds.

### Participants

All consecutive adults aged ≥ 60 years, admitted to general medical wards at the four sites over a period of six months, were invited to participate. Written information regarding the study was read aloud in Swahili by Tanzanian researchers. Following an assessment of capacity, participants provided written informed consent by way of a signature or thumbprint. Those lacking capacity were included if an informant (person aged ≥ 18 years, who knew the patient well, and was not acting in a paid or professional capacity) assented on their behalf. People were excluded if they were less than 60 years of age, refused to participate, or lacked capacity and an informant.

### Measurements

Two frailty instruments that are commonly used, and have been applied in LMICs and SSA previously, were utilised to maximise comparability with existing literature. The first of these was the Clinical Frailty Scale which was developed by Rockwood et al. and is underpinned by a model of frailty as an accumulation of deficits across domains including cognition, function and comorbidity [9]. Though originally conceived as a tool for clinicians to summarise the results of a CGA, the scale has shown great promise as a screening tool for frailty, particularly in acute settings [21]. The second instrument was Fried’s frailty phenotype which characterises physical frailty as a syndrome in which undernutrition, weight loss, reduced strength, poor energy levels and slow-walking speed feed into a downward spiral, resulting in disability and dependency [6].

#### Clinical Frailty Scale

The Rockwood CFS is a 9-item visual screening tool, with supplementary text descriptions, on which a person may be graded from *“1. Very Fit”* to *“9. Terminally Ill”* [9]. The CFS has been applied extensively around the world, including in LMICs, and most frequently in inpatient settings [17, 18, 21]. The CFS was translated verbatim into Swahili and completed by trained researchers. Participants with scores of 1–4 were categorised as non-frail, and those scoring 5–9 as frail for analysis.

#### Frailty phenotype

Variations of Fried’s Frailty Phenotype (FP) have been used extensively throughout the world to assess the prevalence of frailty, both in hospital and in community settings [6, 22]. A version of the FP has previously been successfully operationalised in the community in the Hai District, an area served by the hospitals participating in this study [15]. Based on this previous work by Lewis et al., the present study utilised the FP items and criteria outlined in Table 1.

**Table 1:** Frailty Phenotype items and criteria

Frailty Phenotype Item	Description and frailty criteria
Weak hand grip strength	Hand grip-strength (HGS) was assessed with the participant sat with their arm in 90 degrees of flexion by use of a JAMAR hydraulic hand dynamometer (Model J000105, Lafayette Instruments, Lafayette, IN, USA). The highest reading from a total of three measurements on each side was recorded. The lowest quintile (≤9 kg for males, ≤4kg for females) was used to define the frailty criterion.
Slow walking speed	The time taken for participants to walk 4.5m, at their usual pace and with their usual walking aids, was recorded. The slowest quintile (≥22 seconds) were classified as frail for this item.
Self-reported exhaustion	Participants were read verbatim Swahili translations of the statements *“I felt everything I did was an effort”* and *“I could not get going”* from the Centre for Epidemiological Studies Depression scale (CES-D) [55]. They were asked to give one of a range of prescribed responses considering a usual week, rather than the last week, to avoid the confounding acute illness effect. Frailty criterion were met if participants answered *“a moderate amount of the time”* or *“most of the time”* to either statement.
Weight loss	A verbatim Swahili translation of the question *“Have you lost weight during the last 3 months?”* from the Mini-Nutritional Assessment Short-Form (MNA-SF) was read to participants [51]. Frailty criterion were met if they answered *“Weight loss greater than 3kg”* or *“Weight loss between 1 and 3kg”*.
Low physical activity	Participants were asked a translation of the question “*On how many days do you do moderate physical activities like gardening, cleaning, bicycling at a regular pace, swimming or other fitness activities?”* from the International Physical Activity Questionnaire (IPAQ) [56]. As with the CES-D, participants were asked to consider a usual week, rather than the last week. Frailty criterion were met if participants answered *“0”*.

When originally conceived, assessment using the FP required the older person to be able to walk and have sufficient cognitive faculties to follow instructions for hand grip-strength (HGS) [6]. Due to concerns that this may lead to an underestimation of frailty burden, many studies have since applied the FP without these exclusions, with various strategies for managing missing data [15, 19, 23, 24]. This study applied the FP to all participants and researchers were asked to complete a free text box to justify missing items. It was not possible to use Fried’s original thresholds (where 0 is considered “robust”, 1–2 “pre-frail”, and 3–5 as “frail” [6]) due to a high proportion of missing data. Therefore, participants were only included if they had ≥ 3/5 concordant items (either all positive indicating the presence of frailty, or all negative indicating the absence of frailty). Those with ≥ 3/5 positive items were classified as “frail”, and those with ≥ 3/5 negative items as “non-frail”. This method has previously been used because it avoids the need for any imputation [19]. The main drawbacks are that participants with discordant items cannot be categorised, and for participants with missing items it is often not possible to differentiate “pre-frail” and “robust” categories without imputation, so these groups are combined together as “non-frail” [19].

#### Sociodemographic and clinical characteristics

In addition to frailty screening, sociodemographic and clinical data were recorded including age, sex, marital status, education and literacy. Participants were asked *“What medical diagnoses/conditions did you have before coming to hospital?”* from a list of 16 categories from the Study of Global AGEing and Adult Health Questionnaire (diabetes, hypertension, cataracts, stroke, heart disease, chronic respiratory, tuberculosis, arthritis, stomach bowel of liver problems, dementia, depression, epilepsy, chronic renal failure, cancer, HIV/AIDS or other diagnoses) [25]. The Identifying Dementia in Elderly Africans cognitive screen (IDEA-Cog) [26], IDEA Instrumental Activities of Daily Living tool (IDEA-IADL) [27], Barthel Index [28], EURO-D depression screen [29] and Confusion Assessment Method screen for delirium (CAM) [30] were also completed.

### Statistical analysis

Assessments were inputted into Android tablets using Kobo ToolBox open-access software (Kobo Inc, Cambridge, MA, USA). Data were analysed using IBM SPSS Statistics version 28.0 (IBM, New York, NY, USA). Descriptive statistics are presented as *“mean (± standard deviation [SD])”*, or *“median (± interquartile range [IQR])”* for non-parametric variables. The CFS and FP scores were used to dichotomise participants into frail versus non-frail groups and Pearson’s Chi Squared was used to compare sociodemographic and clinical characteristics in univariate analyses. There was no imputation for missing variables, however Mann-Whitney U and unpaired t-tests were used to compare the CFS scores and clinical characteristics of participants who were classifiable using the FP, against those with insufficient data to determine frailty status; this was conducted to give an indication as to whether missing FP items could be informative (and indicative of frailty), or missing at random. Significance was set at the *p* < 0.05 level, and Exact or Monte-Carlo significance is presented.

## Results

**Fig. 1:**
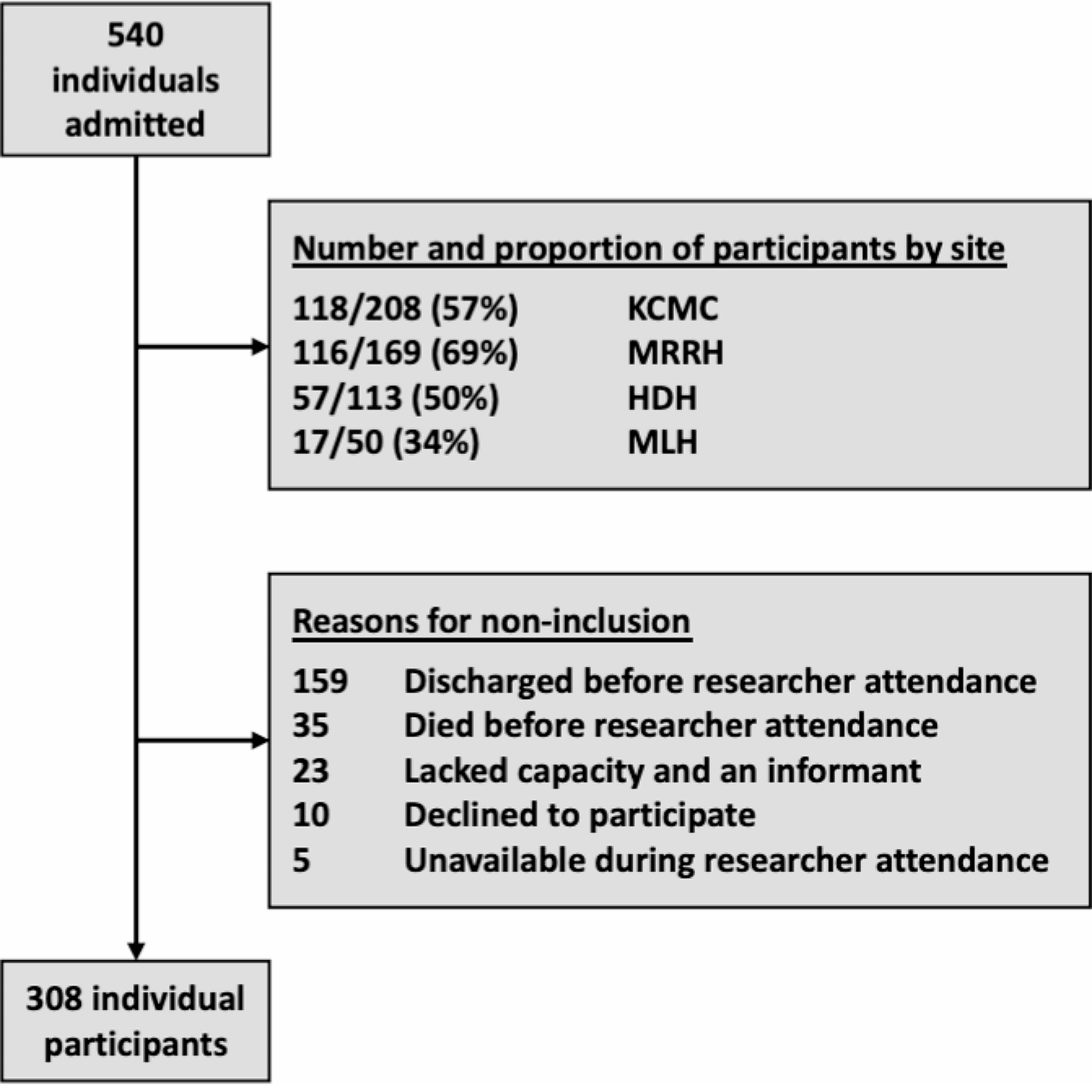
Participation by hospital site and reasons for non-inclusion. *KCMC, Kilimanjaro Christian Medical Centre; MRRH, Mawenzi Regional Referral Hospital; HDH, Hai District Hospital; MLH, Machame Lutheran Hospital*

Between March and August 2022, 540 people aged ≥ 60 were admitted across the four sites. Assessments were completed for 308 individuals, 155 (50.3%) of whom were female and whose mean age was 74.93 (9.92). Figure 1 depicts participation rates for each site and reasons for non-inclusion. Data collection teams were primarily based in the urban sites and had to travel to MLH and HDH. Consequently, the rates of inclusion were lower in these more rural hospitals.

The prevalence of frailty by the CFS and FP is depicted in Table 2. The CFS was completed for all participants, while the FP was only calculable for 149 participants due to a high proportion of missing data.

**Table 2:** The prevalence of frailty by Clinical Frailty Scale and Frailty Phenotype

Frailty screening method	Number of participants (% of total valid)		
	Frail	Non-frail	Total valid
Clinical Frailty Scale	205 (66.6)	103 (33.4)	308
Frailty Phenotype	85 (57.0)	64 (42.9)	149

The sociodemographic and clinical characteristics of frail and non-frail groups are compared in Table 3. Irrespective of which instrument was used to define frailty status, participants with frailty were significantly older, with greater disability and poorer cognitive scores than their non-frail counterparts. Lower levels of education and literacy were also seen amongst the frail groups. Sex and marital status did not significantly differ between groups.

**Table 3:** Sociodemographic and clinical characteristics of participants by frailty status

	Clinical Frailty Scale Number of participants			Frailty phenotype Number of participants		
Characteristic (total N for which this was available)	Frail	Non-frail	Pearson’s	Frail	Non-frail	Pearson’s
			Chi^2^ (sig.)			Chi^2^ (sig.)
	N = 205	N = 103		N = 85	N = 64	
Age (308)						
60–69 years	56	49		26	33	
70–79 years	68	31	**14.446**	25	26	**19.849**
≥80 years	81	23	**(<.001)**	34	5	**(<.001)**
Sex (308)						
Female	108	47	1.364	44	28	0.939
Male	97	56	(0.277)	41	36	(0.408)
Marital status (308)						
Married	99	59	2.217	43	41	2.695
Unmarried*	106	44	(0.148)	42	23	(0.133)
Education (306)						
Secondary or higher	23	22		14	13	
Primary complete	85	58		37	37	
Some primary	26	10	**19.923**	8	7	7.666
No formal	69	13	**(<.001)**	25	7	(0.052)
Literacy (307)						
Read/write well	64	61		38	36	
Read/write with difficulty	46	23	**27.237**	14	20	**13.048**
Unable	94	19	**(<.001)**	32	8	**(<.001)**
Number of self-reported						
chronic conditions (277)						
0–1	87	55	**8.039**	36	33	3.286
≥2	104	31	**(0.006)**	42	20	(0.051)
Barthel Index (308)						
0 ADL disability	14	85	**180.092**	23	49	**55.829**
≥1 ADL disability	191	18	**(<.001)**	62	15	**(<.001)**
IDEA-IADL (301)						
0 IADL disability	93	90	**53.582**	42	57	**30.790**
≥1 ADL disability	108	10	**(<.001)**	42	4	**(<.001)**
IDEA-Cog score (308)						
0–4 (poor cognition)	122	22		40	3	
5–7 (moderate cognition) 8–12 (good cognition)	40 43	23 58	**47.713** **(<.001)**	20 25	16 45	**35.746** **(<.001)**
EURO-D score (303)						
0–3 depressive symptoms	30	24	3.199	7	17	**8.694**
≥4 depressive symptoms	170	79	(0.082)	76	47	**(0.004)**
CAM (293)						
Positive for delirium Negative for delirium	35 157	4 97	**11.678 (<.001)**	10 73	2 60	3.639 (0.071)

*Includes widowed, separated, divorced and unmarried. FP, frailty phonotype; IDEA-IADL Identification of Dementia in Elderly Africans Instrumental Activities of Daily Living screen; IDEA-Cog, Identification of Dementia in Elderly Africans Cognitive screen; EURO-D, depression screen; CAM, Confusion Assessment Method

### Missing data for the frailty phenotype

Missing data meant that the FP could not be calculated for 159 (51.6%) participants. Only 26 (8.4%) completed all five FP items, though 241 (78.2%) were able to complete at least three. The number of participants who completed each FP item, and the reasons for missing items documented by researchers are displayed in Table 4. Compared with all participants whose frailty status could be classified using the FP, the 159 participants with insufficient data were older (76.6 [±10.1] versus 73.1 [±9.4] years, *t* = 3.172, *p* < 0.002), with greater disability indicated by lower scores on the Barthel (6 [±14] versus 17 [±9], *U* = 5673.0, *p* < 0.001) and higher CFS scores (6 [±3] versus 4 [±3], *U* = 6121.0, *p* < 0.001). These and additional clinical characteristics are compared in supplementary Table 1.

**Table 4:** Frailty phenotype items completed and reasons for missing items

				Number of participants (%)	
FP item				Completed item	Did not complete item
	Weak grip strength			158 (51.3)	150 (48.7)
	Slow walking speed			71 (23.1)	237 (76.9)
	Self-reported exhaustion			307 (99.7)	1 (0.3)
	Weight loss			142 (46.1)*	166 (53.9)*
	Low physical activity			307 (99.7)	1 (0.3)
Reasons for missing items					
		Acute illness prevented completion of one or more items		Total General lethargy Hypo/hypertension Stroke/ TIA = 26 Shortness of breath Pain Medical devices Other acute	159 (51.6) 41 (13.3) 27 (8.8) 26 (8.4) 25 (8.1) 12 (3.9) 6 (1.9) 22 (7.1)
		Chronic disability prevented completion of one or more items		Total Previous stroke Chronic immobility Amputation	14 (4.5) 6 (1.9) 5 (1.6) 3 (1.0)

FP, frailty phenotype. *A total of 166 participants answered “Don’t know” to the question “Have you lost weight during the last 3 months?” and and this response was counted as not having sufficient information to complete the item

## Discussion

### Prevalence of frailty

This study indicates that frailty is prevalent amongst older people admitted to hospitals in northern Tanzania. The estimate of prevalence by CFS was strikingly similar to the 63.3% found using the same instrument amongst older medical inpatients in Nigeria [17]. Though comparisons must be drawn cautiously due to the high proportion of missing data, the prevalence using the FP represents a much greater burden of frailty than in the community in Tanzania, where the phenotypic prevalence in the Hai District was 9.3% [15]. This is an expected finding, given that older people living with frailty are more likely to utilise hospital services [31].

Estimates of the prevalence of frailty amongst older hospital inpatients vary widely, not only because of the lack of tools to standardise assessment, but also due to differences in population demographics, culture and healthcare utilisation [32]. A recent systematic review by Doody et al. sought to produce a pooled-prevalence for frailty amongst older people admitted to hospital, including 96 studies which utilised a whole range of frailty instruments across a variety of inpatient settings [32]. Their meta-analysis included more than 460,000 individuals aged 65 years and over, and gave an overall prevalence for frailty of 47.4% [32]. Caution must be applied in making comparisons between this existing literature and the FP results of the present study due to the proportion of missing data. However, Doody et al.’s review included five studies which applied the CFS to unselected admissions on medical and geriatric wards with which some comparison can be made [32]. These gave prevalence estimates from 56.7 to 81%, though it is worth noting all were from HICs [33–37]. The 66.6% found in hospitals in northern Tanzania in this study is within this range and represents a significant burden of frailty for a healthcare system that lacks specialist geriatric care.

### Sociodemographic and clinical associations of frailty

In this study, participants with frailty were comparatively older, with greater disability, higher rates of cognitive impairment and (when status was defined by CFS) significantly greater comorbidity than their non-frail counterparts. These characteristics are consistent with other studies from elsewhere in SSA [15, 17, 19] and other LMICs [38–40]. The relationship between frailty, comorbidity and disability is well-established, though complex, as all three phenomena are highly correlated [41]. Though overlapping, they are distinct entities which can occur in different combinations with variable impact on health outcomes [15, 41]. Consequently, effective care for older patients with frailty must account for this complexity, seeking to balance the treatment of multiple conditions, whilst considering function and independence [13, 41].

Our data underscores the interplay between frailty and cognitive function. Participants with frailty performed significantly worse in cognitive testing and (when defined by CFS) experienced greater rates of delirium than their non-frail counterparts. This is consistent with existing literature that demonstrates a negative relationship between frailty and global cognitive status, as well as greater risk of delirium amongst frail individuals [42, 43]. Education and literacy have the potential to act as confounders in the assessment of cognition. This is a concern in our cohort in which lower literacy levels were seen amongst frail participants. However, it is also worth noting that the IDEA-Cog was developed specifically for use in this setting and did not show any educational bias during validation [26].

In contrast to existing literature, this study found no statistically significant differences between frail and non-frail groups with respect to sex or marital status. Interestingly, levels of frailty also did not differ significantly between sexes in the community in Tanzania, and in a hospital-setting in Nigeria it was in fact males who were at greater risk [15, 44]. This goes against the pattern seen elsewhere, in which women experience higher rates of frailty throughout life despite having longer life-expectancies than men (known as the male-female health-survival paradox) [45]. With respect to marital status, unmarried (and particularly widowed) individuals are usually considered to have a greater risk of frailty, possibly as a result of greater social isolation and vulnerability [46]. This conclusion is based largely on data from “Western” HICs, where nuclear families predominate, and may not be as applicable in Tanzania where extended family structures are still the norm.

### Challenges associated with application of the FP

Though it was possible to apply the CFS to all participants, use of the FP proved challenging in this hospital setting with only 26 (8.4%) participants completing all five items. In community studies in both Tanzania and South Africa, over 80% of participants completed all items [15, 19]. Other studies which have utilised the FP in older people admitted to hospital in LMICs have chosen to exclude non-ambulatory individuals [40, 47]. Though this approach would undoubtably have led to a lower proportion of missing data (particularly with respect to gait speed), this approach also has the potential to miss the frailest individuals. Post-hoc analysis of those participants with insufficient data to be classified according to the FP demonstrated that they were older, with greater disability, worse cognition, and higher scores on the CFS. This echoes findings from the community in South Africa, where higher hazard ratios for frailty were seen amongst individuals with insufficient data to assign a phenotypic category [19], and suggests that missing items may be informative, and indeed indicative of frailty criterion having been met, rather than missing at random.

Symptoms relating to acute illness were the most common reason given by researchers for missing items of the FP and a total of 218 (70.8%) participants reported they could not stand at the time of interview. Amongst studies from HICs that have utilised the FP in the assessment of acute unselected admissions, similar challenges have been seen with respect to measuring walking speed. Rates of completion for this item in studies from the UK and USA respectively range from 30% to 64% [48, 49]. However, the same cannot be said for HGS where in the same studies rates of completion were over 90% and our result is an outlier [48, 49]. This does raise a question as to whether walking speed in this context is truly measuring underlying frailty, or simply a non-specific and potentially reversible effect of acute illness. Potential alternatives that may prove more practical in the LMIC ward setting might include the chair-to-stand test, in which the inability to rise from a chair five times without the use of one’s arms is considered indicative of frailty; this already used as one of the three components of the Study of Osteoporotic Fracture frailty index [50].

Although, a large proportion of participants answered the MNA-SF question regarding weight loss, 53.9% didn’t know if they had lost weight. In SSA access to weighing scales at home and the regular assessment of weight is uncommon. Even in settings where this is not the case, cognitive impairment and delirium can affect the ability of an older person to self-report weight loss with accuracy. Body Mass Index (BMI) is often used as a component of the FP but was not associated with frailty status on univariate analysis in a community setting in Tanzania where concerns were expressed about missing participants with sarcopenic obesity [15]. The full MNA-SF includes BMI, but where this cannot be obtained calf circumference is taken in its place [51]. Calf-circumference is a marker of sarcopenia, which is already utilised in other frailty screening tools, and could provide an alternative measure in the acute hospital setting in future [52, 53].

### Limitations

A limitation of the cross-sectional nature of this study was the non-inclusion of people who died or were discharged prior to researcher attendance, thus potentially missing those who were the most, and the least, frail. Furthermore, lower rates of recruitment from rural sites mean the data are more representative of the experiences of the larger participating hospitals and data were insufficient for subgroup analyses by site.

Though the CFS data are complete, large proportions of missing data for the FP make this a challenge to interpret and to compare to existing literature. By presenting data for only participants with three concordant items, we avoided the need for any imputation. However, this approach excluded those with discordant items who could have been pre-frail or frail if their FP data were complete. This led to an underestimation of the frailty burden by FP. Furthermore, the method does not distinguish pre-frail from robust participants, who are instead grouped together as non-frail. This may have reduced the magnitude of any differences when drawing comparisons to the frail group.

Frailty as a phenomenon can be understood as a quality, a process or a construct, which has evolved in the socio-cultural context of “Western” biomedicine [54]. Even within this context, there is diversity in its understanding and in how it is operationalised. This study did not interrogate social constructs or physiological factors and utilised tools which were designed for use in a “Western” context. Qualitative exploration of healthy ageing in Tanzania also highlights the importance of social determinants not covered by these tools, especially with respect to relationships with community and financial resources [16].

## Conclusions

This research indicates that the prevalence of frailty amongst older people admitted to medical wards in northern Tanzania is high and that people with frailty in this context were older, with lower literacy, greater disability and poorer cognitive performance. It was possible to apply the CFS to all participants, though the FP proved challenging and demonstrates the need for adaption of tools for an African social context. Future work should also seek to correlate frailty status with long-term clinical outcomes for older people after admission in this setting.

### Electronic supplementary material

Below is the link to the electronic supplementary material.

Supplementary Material 1

## Data Availability

The data are available from the corresponding author on request.

## Abbreviations

ADL: Activities of Daily Living
CAM: Confusion Assessment Method
CFS: Clinical Frailty Scale
FP: Frailty Phenotype
LMIC: Low- and Middle-Income Country
HDH: Hai District Hospital
HGS: Hand grip-strength
HIC: High-Income Country
IADL: Instrumental Activities of Daily Living
IDEA-Cog: Identification of Dementia in Elderly Africans Cognitive screen
IDEA-IADL: Identification of Dementia in Elderly Africans Instrumental Activities of Daily Living screen
IQR: Interquartile range
KCMC: Kilimanjaro Christian Medical Centre
MNA-SF: Mini-Nutritional Assessment Short-Form
MRRH: Mawenzi Regional Referral Hospital
MLH: Machame Lutheran Hospital
SSA: Sub-Saharan Africa
SD: Standard deviation
