# Supplementary material for: Assessing frailty amongst older people admitted to hospital in a low-income setting: a multicentre study in northern Tanzania
Source: BMC Geriatr. 2024 Feb 26;24:190. doi: 10.1186/s12877-024-04789-6 (PMC10898155; doi:10.1186/s12877-024-04789-6)
Supplement: Supplementary file 1 — Supplementary Material 1 [file 12877_2024_4789_MOESM1_ESM.docx]

**Supplementary table 1. Comparison of participants with sufficient data for FP classification versus those with insufficient data**

|  | Sufficient data for FP (N=149) | Insufficient data for FP (N=159) | Test statistic | Sig. |
| --- | --- | --- | --- | --- |
| Mean age in years (SD) | 73.1 (9.4) | 76.6 (10.1) | 3.172 | **<.002** |
| CFS median score (IQR) | 4 (3) | 6 (3) | 6121.5 | **<.001** |
| IDEA-cog median score (IQR) | 7 (6) | 2 (7) | 6922.0 | **<.001** |
| IDEA-IADL median score (IQR) | 16 (20) | 29.5 (27.5) | 7225.5 | **<.001** |
| Barthel Index median score (IQR) | 17 (9) | 6 (14) | 5673.0 | **<.001** |

*FP, frailty phenotype; SD, standard deviation; IQR, interquartile range.*
